# Nitrosylation vs. oxidation – How to modulate cold physical plasmas for biological applications

**DOI:** 10.1371/journal.pone.0216606

**Published:** 2019-05-08

**Authors:** Jan-Wilm Lackmann, Giuliana Bruno, Helena Jablonowski, Friederike Kogelheide, Björn Offerhaus, Julian Held, Volker Schulz-von der Gathen, Katharina Stapelmann, Thomas von Woedtke, Kristian Wende

**Affiliations:** 1 ZIK *plasmatis* at Leibniz Institute for Plasma Science and Technology (INP Greifswald e.V.), Greifswald, Germany; 2 Institute for Electrical Engineering and Plasma Technology, Ruhr University Bochum, Bochum, Germany; 3 Experimental Physics II, Ruhr University Bochum, Bochum, Germany; 4 Plasma for Life Sciences, Department of Nuclear Engineering, North Carolina State University, Raleigh, North Carolina, United States of America; Universite Toulouse III Paul Sabatier, FRANCE

## Abstract

Thiol moieties are major targets for cold plasma-derived nitrogen and oxygen species, making CAPs convenient tools to modulate redox-signaling pathways in cells and tissues. The underlying biochemical pathways are currently under investigation but especially the role of CAP derived RNS is barely understood. Their potential role in protein thiol nitrosylation would be relevant in inflammatory processes such as wound healing and improving their specific production by CAP would allow for enhanced treatment options beyond the current application. The impact of a modified kINPen 09 argon plasma jet with nitrogen shielding on cysteine as a thiol-carrying model substance was investigated by FTIR spectroscopy and high-resolution mass spectrometry. The deposition of short-lived radical species was measured by electron paramagnetic resonance spectroscopy, long-lived species were quantified by ion chromatography (NO_2_^-^, NO_3_^-^) and xylenol orange assay (H_2_O_2_). Product profiles were compared to samples treated with the so-called COST jet, being introduced by a European COST initiative as a reference device, using both reference conditions as well as conditions adjusted to kINPen gas mixtures. While thiol oxidation was dominant under all tested conditions, an Ar + N_2_/O_2_ gas compositions combined with a nitrogen curtain fostered nitric oxide deposition and the desired generation of S-nitrosocysteine. Interestingly, the COST-jet revealed significant differences in its chemical properties in comparison to the kINPen by showing a more stable production of RNS with different gas admixtures, indicating a different ^•^NO production pathway. Taken together, results indicate various chemical properties of kINPen and COST-jet as well as highlight the potential of plasma tuning not only by gas admixtures alone but by adjusting the surrounding atmosphere as well.

## Introduction

Cold atmospheric plasmas (CAP) are used in a wide variety of fields. In particular, the interest in medical applications has increased in recent years. Multiple scientific studies and case studies confirm CAPs effectiveness for therapeutic purposes, such as wound healing and skin regeneration but also cancer treatment [1–3]. Plasma sources have different designs and discharge concepts, and consequently vary in gas composition and chemical properties of the produced plasma [4–6]. In particular, various reactive oxygen and nitrogen species (RONS) are deposited in treated liquids or in the cellular environment. Production of these species in the gas phase and interaction at the gas-liquid interface vary according to the chosen plasma source and related parameters, leading to a distinct deposition of RONS in the liquid bulk [7–9]. In biological systems, these plasma-generated species modulate redox-signaling processes, ultimately leading to functional consequences [10]. Among these, an increased expression of anti-oxidant proteins such as members of the glutathione metabolism, changes in cell migration rate and cell viability have been observed in cell [11] and animal models [12]. Given the fact, that most of the plasma-derived species are short lived, the question arises which biochemical mechanisms are relevant to relay the chemical information from the plasma to the cell. Several studies focus on the deposition and production of RONS in liquid media for biomedical applications. Chauvin *et al*. investigated several media after treatment with a plasma jet and demonstrated efficient deposition of both long (hydrogen peroxide, nitrite, and nitrate) and short-living (superoxide, hydroxyl radicals) species [13]. Furthermore, a possible pathway is the (covalent) modification of biomolecules, *e*.*g*. at the amino acid cysteine. Its thiol group can bear oxidation states from -2 to +6, forming a number of chemotypes with biological importance [14]. An initial oxidation product is cysteine sulfenic acid (RSOH). RSOH rapidly reacts with other thiols to form disulfides (RSSR), such as cystine. Strong oxidizing agents can lead to a progressive oxidation of cysteine to its sulfinic (RSO_2_H) and/or sulfonic acids (RSO_3_H) [14, 15]. In mammalian cells, this reactivity is harnessed in redox signaling processes were an initial step in signal transduction is the controlled oxidation of cysteines, *e*.*g*. in peroxiredoxins, that subsequently lead to changes in protein conformation, trafficking, or downstream chemical processes (“thiol switches”) [16–18]. Other biologically active modifications are S-glutathionylation and S-nitrosylation, modulating the cysteine residue activity and protein function [19, 20]. Comparable covalent modifications, especially oxidations, have been reported for the reaction of cold plasma with thiol groups, leading to the formation of disulfides, partially oxidized disulfides, and cysteine-thiooxo acids. Besides thiols, other protein compounds were also shown be modified by CAP treatment, such as phenylalanine and proline [21] as well as histidine, methionine, and tryptophan [22]. Predominantly reactive oxygen species have been attributed as plasma-derived precursors fostering these reactions [3, 17, 23–28]. Despite the considerable abundance of nitric oxides (NO^•^, N_2_O_3_, ^•^NO_2_) and other RNS in the gas phase of many plasma sources, little is known about the role in plasma liquid chemistry. Plasma derived RNS contribute to oxidative processes but presumably lead to the formation of nitrosylated products, such as S-nitrosocysteine. Physiologically, thiols play a key role in the nitric oxide pathway as sink and activity modulator.

S-nitrosocysteine occurs during inflammation processes and defense responses, *e*.*g*. against pathogens or cancerous cells [29–31]. Thiol nitrosylation protects proteins during oxidative stress by scavenging ^•^NO_2_ and N_2_O_3_, and by avoiding the formation of irreversible sulfur oxidation procured by oxygen derivatives such as O_2_^•-^, ^•^O and O_3_ [19, 32]. However, the erroneous production of S-nitrosothiol has been associated with cancer development as well as with pulmonary, cardiac, and neurological pathologies [33, 34]. S-nitrosothiols are considered as efficient ^•^NO-donors acting as substrates for various ^•^NO-producing enzymes, and their action as anticancer agents has been proven both *in vivo* and *in vitro* applications [35–38]. Therefore, the deposition of ^•^NO by cold plasmas and the potential formation of nitrosylated thiols is of great interest for their (bio-)medical application.

In this study, we investigate the impact of two different plasma sources, the kINPen [39] and the COST-jet [40], on cysteine model solutions, in order to investigate the production of S-nitrosocysteine. The structure, origin, and kinetic profiles of the resulting cysteine derivatives were determined using FTIR spectroscopy and time-of-flight high-resolution mass spectrometry. At the same time, the production of reactive oxygen and nitrogen species in the treated solutions has been studied using spin trap enhanced electron paramagnetic resonance spectroscopy (EPR, for liquid phase ^•^NO), a colorimetric assay (for H_2_O_2_) and ion chromatography (for NO_2_^-^ and NO_3_^-^).

## Materials and methods

### Plasma sources

The kINPen 09 (kINPen; neoplas GmbH) and the COST Reference Microplasma Jet (COST-jet) were investigated in parallel. The kINPen consists of a high-voltage needle electrode located in the center of a ceramic capillary having a 1.6 mm diameter. A radio frequency of around 1 MHz drives the needle electrode, with an averaged power of about 1.1 W. For the experiments, a gas flow rate of 3 standard liters per minute (slm) [41, 42] was kept constant and either pure argon was used or 1% of the feed gas was replaced with N_2_ or O_2_. Alternatively, a mixture of 0.3% N_2_ and 0.7% O_2_ (all gases at 5.0 purity or 4.8 for O_2_, all gases purchased from Air Liquide) was used. The distance of the jet nozzle to the liquid surface was set to 9 mm for all experiments. The atmosphere has an impact on the plasma-induced chemistry for jets where the plasma propagates out of the nozzle. While the COST-jet is less affected as the discharge is confined between the electrodes, the kINPen can be influenced as the discharge propagates out of the nozzle. To allow for more stable ambient conditions in case of the kINPen, an external curtain gas consisting of N_2_ with a flow rate of 5 slm was applied. The curtain gas device is formed by a concentric channel, which outlet was located exactly 1.9 mm from the jet’s nozzle. It was shown that the presence of a curtain gas during the treatments influences the production of reactive species resulting in a different interaction with the treated fluids in which they are deposited [43–47].

The COST-jet is the outcome of the European COST action MP 1101 “Biomedical Applications of Atmospheric Pressure Plasma Technology” (2011–2015). It is intended as a general “baseline” onto which the efficacy of other plasma sources can be compared. It consists of two 1 mm thick metal plate electrodes spaced 1 mm apart in between the plasma is ignited. Two quartz glass windows make up the other sides of the rectangular gas channel, resulting in a plasma volume of 30 mm^3^. The plasma is ignited capacitively and is driven by an AC voltage at a frequency of 13.56 MHz. For further details see Golda *et al*. [40]. COST-Jet treatments were facilitated at a sample distance of 4 mm with the discharge power held constant at about 300 mW and a total gas flow of 1 slm. For gas mixtures, pure helium was used as well as the COST-jet “baseline” condition using an admixture of 0.5% O_2_. Additionally, to allow a closer comparison to the kINPen, admixtures of 1% O_2_, 1% N_2_ or a mixture of both gases (0.3% N_2_ + 0.7% O_2_) were used. For all experiments with the exception of the EPR measurements, the COST-jet setup featured an additional cold trap using dry ice to purge the feed gases from any additional water residues and all gases were of 5.0 purity (Linde). Due to the short-lived nature of radicals, even spin-trapped ones, the COST-jet was transported to the INP for EPR measurements. Therefore, no cold trap was used for these experiments and O_2_ admixture was of 4.8 purity (Air Liquide).

### Sample preparation & plasma treatments

The cysteine model solutions were prepared daily by dissolving crystalline cysteine (Sigma-Aldrich) in double-distilled water (ddH_2_O, Millipore) for a final concentration of 300 μM before each analysis, in order to minimize cysteine spontaneous oxidation. A 750 μL volume of each solution was treated in 24-well plates, with a 9 mm distance from the kINPen and 4 mm from the COST-jet, respectively. Prior to treatments, the well plates were cleaned with methanol in order to remove all kinds of compound that might interfere with downstream analytics. Treated samples were divided into aliquots and subjected to subsequent analysis.

Based on previous experiments, certain general treatment conditions were chosen for all of the following experiments. For both sources, conditions were chosen, which are already well investigated for their physio-chemical properties, e.g. production of certain RONS. Such groundwork is essential when trying to understand the impact of plasma-generated species onto biological targets. The COST-jet was employed using the reference conditions described in [40], as here, most information about RONS fluxes are known. The same is true for the chosen gas compositions. Using the COST-jet in this way allows other research groups to compare and adjust their own experiments to the data presented here using the COST-jet as a reference in the same way by performing experiments once with their own source and once with a COST-jet source. Further gas variations were chosen for the COST-jet to mimic molecular gas admixtures used with the kINPen. For the kINPen, standard conditions were chosen as well as these offer the most information to allow for data interpretation. In previous experiments, 1% of molecular gas admixtures were shown to yield a good balance between species production and discharge stability. Besides pure molecular gas admixtures, one condition was chosen where previous studies could demonstrate a high production of ^•^NO radicals [48]. Furthermore, using 3 slm total gas flux at a distance of 9 mm was investigated in detail as well. These conditions allow treatments of liquids in 24 well plates without pushing the liquid to the well walls, which would result in the treatment of the well bottom instead of the liquid. Furthermore, the curtain gas setup was chosen for the kinpen as its discharge propagates out of the nozzle into the air and previous experiments have shown the strong impact of ambient conditions [49]. The COST-jet is less affected by ambient air due to its discharge being completely encased in its housing. Treatment times of 30 s were chosen after previous experiments showed a strong degradation of labile cysteine compounds at longer treatments [50]. For example, one product of high interest, S-nitrosocysteine, is known to be relatively unstable. Therefore, treatment times were further reduced compared to previous publications to increase yield of such unstable but potentially highly valuable products. However, to indicate general behavior of product formation over time, mass spectrometry experiments were performed for several time points (5 s, 15 s, 30 s, 45 s, 60 s) and resulting products assessed.

### FTIR spectroscopy

For FTIR spectroscopy of cysteine silicon wafers (Siltronic AG) were chosen as substrate material, because of their transmission in the desired wavelength range. Wafers were cut into pieces (approx. 10 mm × 10 mm) and rinsed with ethanol prior to sample preparation. Droplets of 10 μl (COST-jet) or 15 μl (kINPen), respectively, of the plasma-treated cysteine model solutions were pipetted on the silicon wafers and dried by desiccation. A Bruker VERTEX FTIR-micro spectrometer was used for analysis of the L-cysteine samples according to Kogelheide *et al*. [26] The FTIR spectra were recorded from 750 cm^-1^ to 4000 cm^-1^ with a spectral resolution of 4 cm^-1^ and a special focus on the free thiol signal (ν(S-H)) at 2545 cm^-1^. For each droplet of cysteine, 10 spectra at different positions of the sample were recorded with each spectrum representing the mean of 64 measurements at each given position. Background spectra were taken for each measurement to correct the influence of carbon dioxide and water in certain regions of the spectra. All transmission spectra (T) are converted into absorption spectra (A) using the relation

$$A=log\left(\frac{1}{T}\right)$$

and baseline correction is carried out. Normalizing of the corrected data are carried out as follows:

$$x_{i,norm}=\frac{x_{i}}{∥x∥},$$

where ‖x‖ denotes the Euclidean norm, x_i_ is the data point of wavenumber i and x denotes the vector of all sample points. The standard deviation was calculated assuming a Student’s t-distribution. The relative peak intensities were calculated by integrating the peaks and subtracting the corresponding peak intensity, which is taken from the control sample.

### Mass spectrometry

To elucidate the cysteine modifications caused by plasma treatment, high-resolution mass spectrometry (MS) was performed using a TripleTOF 5600 system (Sciex). Isotopically labelled cysteine (L-Cysteine-^13^C_3_^15^N; Sigma-Aldrich, #658057) served as internal standard (IS) at a fixed concentration of 100 μM. It was mixed with the sample directly before the emitter tip of the mass spectrometer through a capillary mixing tee using an electronically controlled syringe pump to prevent long-lived reactive species-derived modifications to the IS before analysis. Each sample was diluted 1:1 with 0.3% ammonium hydroxide in methanol (both Sigma, MS grade), to improve negative electrospray ionization. A flow of 10 μL·min^-1^ of each solution was injected into the Turbo V electrospray source (Sciex), using the following experimental parameters: capillary temperature 150°C, curtain gas: 35 slm N_2_, ion source gas 1: 20 slm N_2_, ion source gas 2: 25 slm N_2_, ion spray voltage: -4000 V.

Survey spectra acquisition (MS) was performed in a 50–400 m/z range and with negative polarity (accumulation time 250 ms). Fragmentation spectra (MS/MS) of each identified peak were acquired in product ion mode (collision energy -24 eV, declustering potential -10 kV) in order to obtain structural information and to identify the cysteine derivatives following each kind of treatment. The formula of each compound and the accurate mass have been identified through the support of the "Formula Finder" and "Mass Calculator" tools included in PeakView 1.2.0.3 (Sciex). For data analysis, a threshold value of 300 counts of the peak height (15 times background) was set for the integration of the total ion current (TIC) signals to ensure proper signal-to-noise ratio. Calibration of the mass spectrometer was performed daily against the mass-to-charge ratios of cysteine and its most representative derivative products. Cysteine-free double-distilled water solutions and cysteine solutions with H_2_O_2_ were used as control solutions for subsequent analyses and data evaluation. For data analysis, the area of each identified peak in a spectrum was first normalized on the total area of each spectrum (area percent) to adjust peak areas for variances in injection. Afterwards, the area percent of each identified molecule was normalized to that of the IS to adjust for variation in ionization efficacy and spray stability.

### Electron paramagnetic resonance spectroscopy

For the detection of ^•^NO in solution, electron paramagnetic resonance (EPR) spectroscopy measurements were performed. An X-band (9.87 GHz) spectrometer (EMXmicro, Bruker) was used with a modulation frequency of 100 kHz, modulation amplitude of 0.1 mT, microwave power of 5.024 mW, receiver gain of 30 dB, and time constant of 0.01 ms. To detect ^•^NO, a nitronyl nitroxyl radical (NNR) spin trap (Carboxy-PTIO (2-(4-Carboxyphenyl)-4,4,5,5-tetramethylimidazoline-1-oxyl-3-oxide, Dojindo Laboratoire), was used at 60 μM, which forms an imino nitroxyl radical (INR) with ^•^NO. BMPO (5-tert-Butoxycarbonyl-5-methyl-1-pyrroline-N-oxide, also Dojindo) was used as a spin trap for sulfur, hydroxyl, and superoxide anion radicals. For some experiment, 60 μM Carboxy-PTIO were mixed with 2 mM BMPO immediately before use. The spin trap solutions were freshly prepared prior each experiment and untreated samples were measured as respective controls. The maximum handling delay between treatment and measurement was three minutes. The spectra were recorded and evaluated by Bruker’s Xenon software. More details about the measurement procedure and evaluation of the spectra can be found in a previous publication [47].

### Ion chromatography

The quantification of nitrite (NO_2_^-^) and nitrate (NO_3_^-^) was performed via ion chromatography (ICS-5000, Thermo Fisher Scientific). After treatment, samples were diluted three fold using ultrapure water (MilliQ) before injecting 10 μL onto an IonPac AS23 anion exchange column (2 x 250 mm, Thermo Fisher Scientific). An isocratic mobile phase (4.5 mM Na_2_CO_3_/0.8 mM NaHCO_3_) with 250 μl/min flow rate was used. Chromatographic data was collected using a combined UV (210 nm) and conductivity detector. The system was calibrated using the Dionex 7-anions standard (Thermo Fisher Scientific) on a weekly basis.

### Colorimetric hydrogen peroxide assay

Hydrogen peroxide (H_2_O_2_) was quantified via the colorimetric reaction with xylenol orange using a commercially available assay (Pierce Quantitative Peroxide Assay Kit, Thermo Scientific) according to the manufacturer’s protocol. The assay is based on the oxidation of ferrous to ferric ion by hydrogen peroxide in presence of the dye which can be detected photometrically (Tecan Infinite M200 Pro, Tecan) at 595 nm. Each 96-well plate contained a standard curve (0 to 150 μM, in triplicates) and the samples (n = 4, each sample in triplicates).

## Results

### First screening of appropriate gas variations

**Fig 1:**
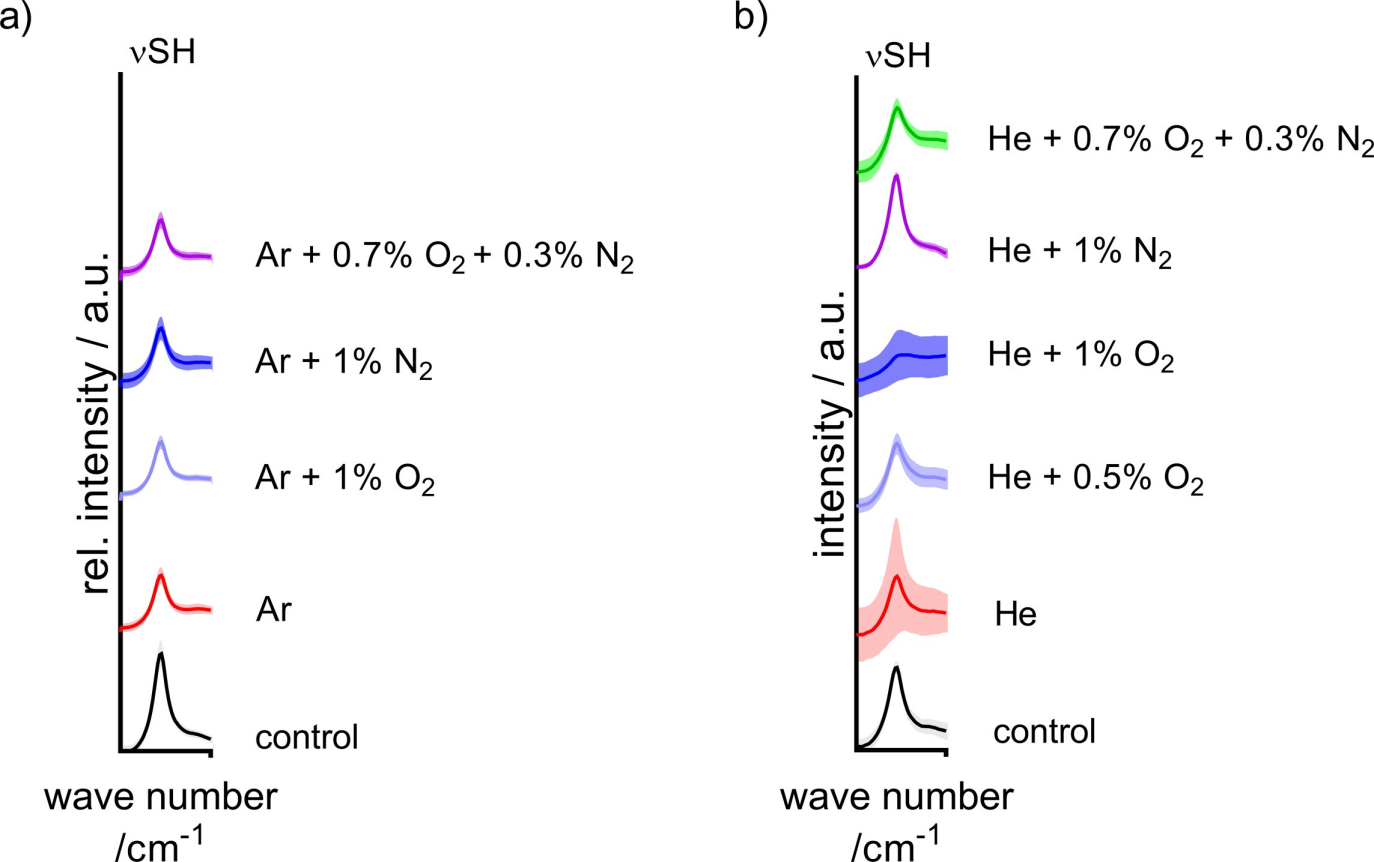
Thiol band signal of cysteine after plasma treatment. The FTIR signal at 2545 cm^-1^ was observed as an indicator of free thiols in the plasma-treated samples. The ν(S-H) band after 30 s of kINPen (a) and COST-jet (b) treatment used for rapid screening to identify plasma conditions with the highest impact on cysteine. Means are shown (lines) with shadowed area denoting standard errors calculated from all respective measurements (n = 3×64 spectra). Peak annotation following Pawlukojc *et al*. [51].

In a first step, the general impact of plasma treatment on the cysteine probe was examined. From previous publications, it could be expected that the thiol moiety would be the most susceptible target of plasma-generated species [50]. As a quick first screening, the ν(S-H) band (2545 cm^-1^) was observed for all treatment conditions (Fig 1). A decrease of this band indicates a loss of free thiol moieties in the sample, suggesting weak or strong interaction of plasma-generated species with the thiol group, respectively. Several conditions were screened and the most promising variations are presented here and used for further study. kINPen treatment showed a notable influence on the thiol group regardless of gas composition. Ar + O_2_ or the combined O_2_ + N_2_ mixture show a slightly stronger reduction of the thiol band as the other conditions. For the COST-jet, oxygen admixtures, especially 1% O_2_ showed a very strong effect on ν(S-H) intensity, whereas treatments using either N_2_ or pure He were least effective in thiol conversion.

### Determination of cysteine derivatives after plasma treatment

**Fig 2:**
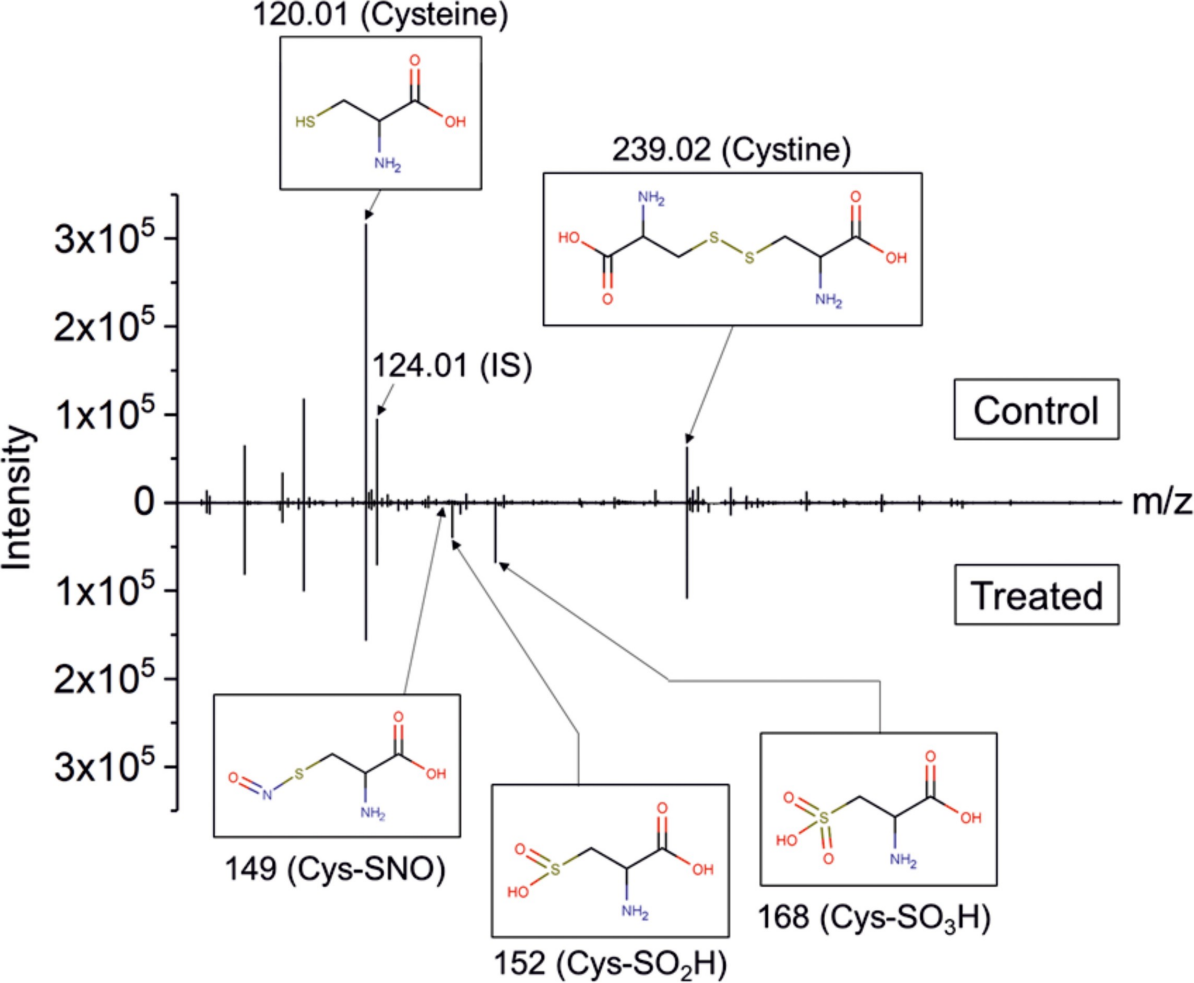
Representative MS spectra of untreated control and kINPen-treated cysteine solutions. Cysteine was treated with kINPen for 30 s using Ar with 0.3% O_2_ + 0.7% N_2_ as working gas and 5 slm N_2_ as curtain gas. The peak at 124.02 m/z represents the internal standard (IS). Selection of identified peaks: cysteine (120.01 m/z), cystine (239.02 m/z), S-nitrosocysteine (Cys-SNO, 149 m/z) (not visible in the spectra), cysteine sulfinic acid (Cys-SO_2_H, 152 m/z), cysteine sulfonic acid or Cys-SO_3_H (168 m/z).

Mass spectrometry was used to determine the observed cysteine modifications at different treatment times with a special focus on nitrosylated cysteine derivatives. Table 1 shows details of the identified major compounds for easier reference. In Fig 2, a representative spectrum recorded for untreated and treated cysteine solution is shown. The structures of the different cysteine derivatives were identified by MS/MS analysis of signals of interest and respective fine masses.

**Table 1:** Major compounds, formula, and mass to charge value of identified compounds before and after cysteine treatments with CAP.

Compound	Formula	m/z
Cysteine	C_3_H_7_NO_2_S	120.0119
Cysteine-^13^C_3_, ^15^N (IS)	C_3_H_7_NO_2_S	124.0191
Cystine	C_6_H_12_N_2_O_4_S_2_	239.016
Cysteine sulfinic acid	C_3_H_7_NO_4_S	152.0017
Cysteine sulfonic acid	C_3_H_7_NO_5_S	167.9967
*S*-nitrosocysteine	C_3_H_6_N_2_O_3_S	149.0021

**Fig 3:**
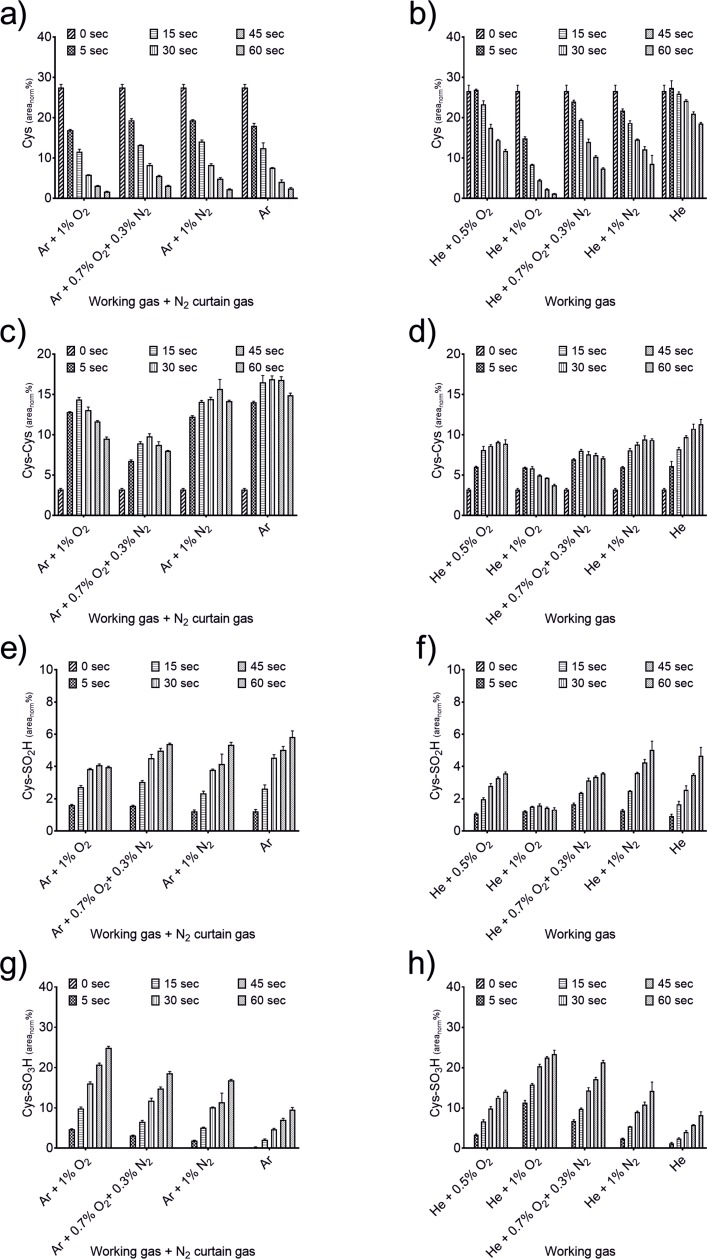
Relative abundances of cysteine and produced oxidative derivatives after plasma treatment. Either the kINPen with N_2_ curtain gas (a, c, e, g) or COST-jet (b, d, f, h) were used for treatments of different durations (5, 15, 30, 45, 60 s) with the denoted gas variations. Spectra were normalized on the IS and peak areas were calculated. Triplicates (n = 3) with standard deviation are shown.

**Fig 4:**
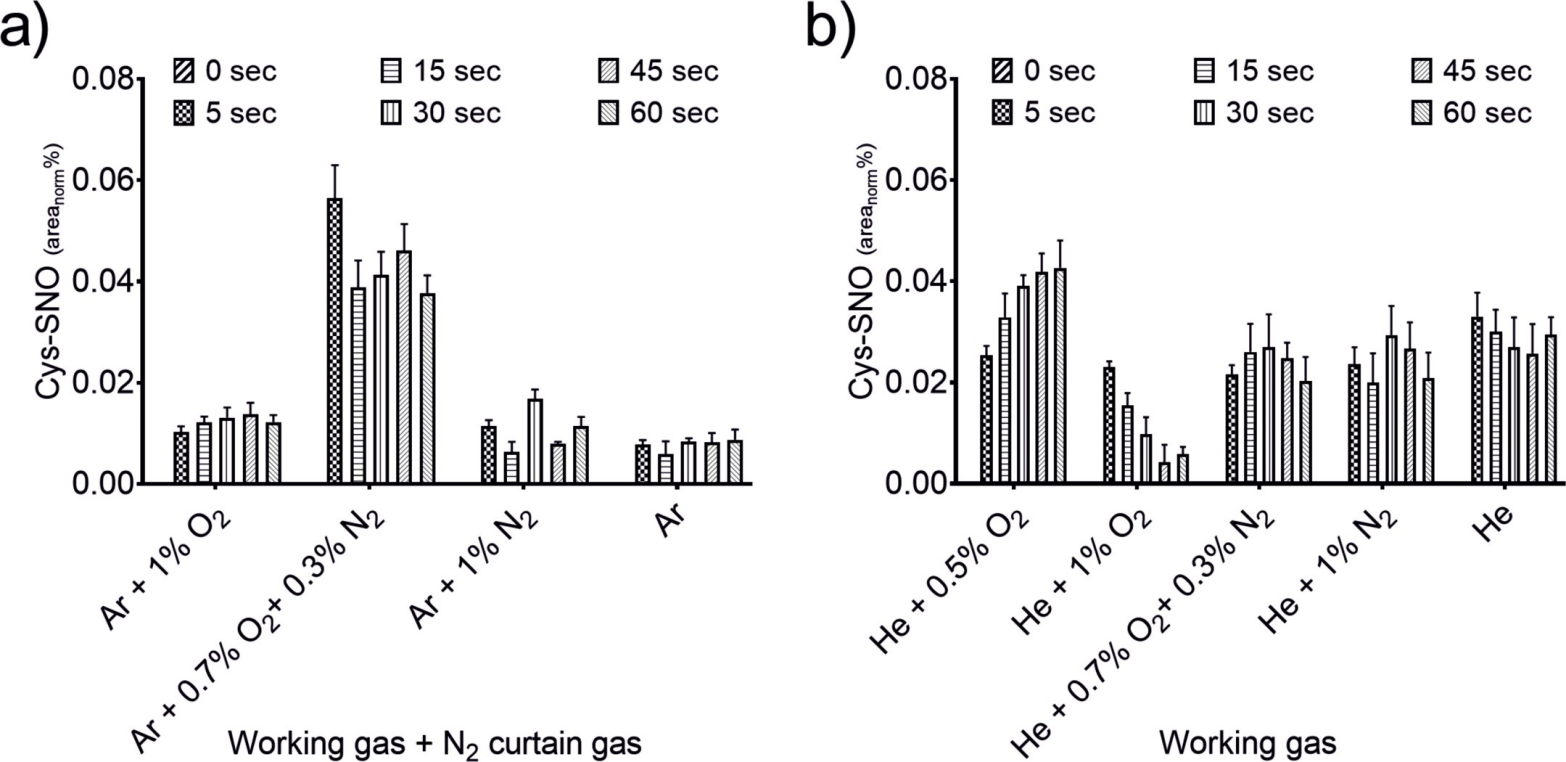
Production of Cys-SNO due to plasma treatment. Either kINPen with N_2_ curtain gas (a) or COST-jet (b) were used for treatments of different durations (5, 15, 30, 45, 60 s) with the indicated feed gas variations. Spectra were normalized on the IS and peak areas were calculated. Triplicates (n = 3) with standard deviation are shown.

As shown in Fig 2, the dominant changes observed after kINPen treatment were of oxidative nature and predominantly concerned the thiol structure. Cystine (C_6_H_11_N_2_O_4_S_2_, 239.0160 m/z) and cysteine sulfonic acid (Cys-SO_3_H, C_3_H_6_NO_5_S, 167.9967 m/z) were the most abundant compounds, followed by cysteine sulfinic acid (Cys-SO_2_H, C_3_H_6_NO_4_S, 152.0018 m/z), which is in good agreement with previous experiments [17]. The initial product of these derivatives, cysteine sulfenic acid (Cys-SOH, C_3_H_6_NO_3_S, 136.0068 m/z), was not observed, most likely due to its instability during ionization. To allow a more comprehensive overview of the production efficacies of the various sources and conditions, spectral intensities were normalized on the spiked-in IS (heavy cysteine, ^13^C_3_H_7_^15^NO_2_S, 124.02 m/z) and the normalized peak areas plotted for the different identified products (Fig 3 and Fig 4). Treatments with the kINPen (Fig 3A, 3C, 3E and 3G) showed the highest consumption of cysteine (Fig 3A) and subsequent production of oxidized stable derivatives, such as cysteine sulfonic acid (Fig 3G), were observed for 1% O_2_ admixture and prolonged treatment times. Under the same conditions, labile derivatives, such as cystine (Fig 3C) and cysteine sulfinic acid (Fig 3E), decreased over time. In contrast, admixture of either 1% N_2_ or the O_2_-N_2_ mixture led to fewer stable oxidized products, combined with an increase of labile compounds, showing a lower oxidative impact.

Treating the cysteine solution with the COST-jet led to the formation of a largely similar product range and yields (Fig 3B, 3D, 3F and 3H). Indeed, by using 1% O_2_ in He working gas, an even higher cysteine oxidation was achieved (Fig 3B). A strong production of sulfonic acid was observed (Fig 3H), while cystine (Fig 3D) and sulfinic acid (Fig 3F) decreased. As observed for the kINPen treatment, working gas with lower amounts of oxygen resulted in a lower oxidative impact on cysteine and its chemical relatives. The observed product range clearly indicate a major role of reactive oxygen species in CAP-derived liquid phase chemistry of both plasma sources investigated. It is not manifest, which species play the most dominant role, though atomic oxygen and singlet oxygen must be considered because oxygen-enriched CAPs produce these species in high amounts [8, 52, 53]. Conditions where predominantly hydrogen peroxide is deposited (pure Ar or Ar + 1% N_2_), were found less effective in the production of higher oxidized cysteine derivatives. Similarly, a limited oxidation of cysteine is evident in conditions of low or non-oxygen admixtures in the feed gas, such as pure helium, by the predominant production of cystine (Fig 3E and 3F).

Although oxidative modifications of the cysteine’s thiol moiety were dominant S-nitrosocysteine (Cys-SNO, C_3_H_6_N_2_O_3_S, 149.0021 m/z) was found in small quantities, indicating the presence and direct reaction of plasma-derived nitrogen reactive species (Fig 4). For the kINPen (Fig 4A), modulation of the working gas had a strong impact on Cys-SNO production. While admixture of oxygen quenched its production, treatment of the cysteine solutions with pure Ar as well as N_2_ or N_2_ + O_2_ admixtures resulted in a significantly higher production. In contrast, relatively stable concentrations were observed for all COST-jet conditions in ambient air environment (Fig 4B). Interestingly, it became obvious that the implementation of the curtain gas setup yielded a higher Cys-SNO production than using the kINPen in ambient air [50]. As production of oxidative modifications were in good agreement between both sources, the variations in Cys-SNO production were further investigated to reveal the related liquid chemistry occurring during plasma treatment.

### Elucidation of free radicals in cysteine-containing solutions

**Fig 5:**
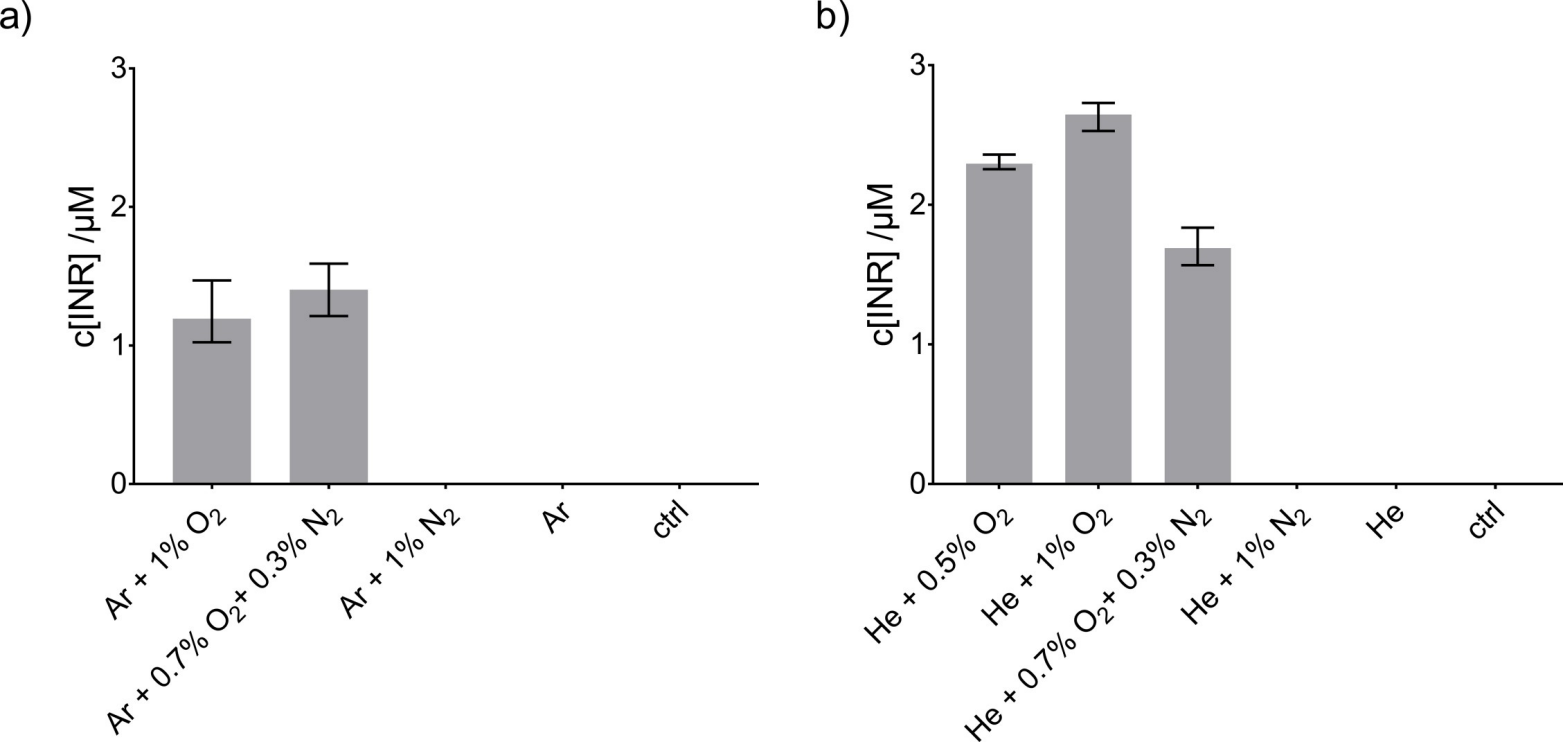
Concentration of the ^•^NO-spin trap adduct (INR) after treatment of ddH_2_O. Treatment was performed with the kINPen in N_2_ environment (a) using the curtain gas device for 9 mm distance between the nozzle and the liquid surface or the COST-jet (b) at 4 mm nozzle-liquid distance in atmospheric air environment. For both plasma sources, different feed gas compositions were studied. Triplicates (n = 3) with standard deviation are shown.

Deposition of free radicals was analyzed for both sources using EPR spectroscopy. In Fig 5, the resulting concentrations of the ^•^NO-adduct (INR, imino nitroxide) are given for the different feed gas compositions after kINPen (a) or COST-jet (b) treatment of ddH_2_O. No clear difference could be determined for feed gas admixture containing either 1% O_2_ or 0.7% O_2_ + 0.3% N_2_ (Fig 5A). In cases where no O_2_ was present in the feed gas (Ar or Ar + 1% N_2_) as well as for the gas control (Ar + 0.3% N_2_ + 0.7% O_2_ without plasma ignition) no ^•^NO-adduct was measured. This is in agreement with a previous study, where the origin of the liquid phase ^•^NO was investigated [54]. There, the optimum of ^•^NO production in phosphate buffered solution has been found to be at O_2_ amounts of 0.7% O_2_ + 0.3% N_2_ in the argon feed gas using N_2_ as curtain gas. A lower amount of O_2_ in the feed gas resulted in a lower ^•^NO-adduct concentration in the liquid. Moreover, a small amount of N_2_ in the feed gas increased production. Details about possible formation pathways of ^•^NO in the liquid can be found in Jablonowski *et al*. [54].

The deposition of NO by the COST-jet was in the same concentration range with highest amounts determined forHe with 0.5% and 1% O_2_ admixture. When the feed gas contained both O_2_ and N_2_, yields decreased.

To meet reference conditions, the COST-jet was operated at 4 mm electrode-liquid distance, compared to 9 mm for the kINPen., which might result in higher ^•^O densities near the gas-liquid interface for the COST-jet. However, ^•^O densities can also be affected by the mean gas velocity, which is higher for kINPen, as well as the gas flux dynamics (turbulent in kINPen, laminar in COST-jet) [49, 55]. The COST-jet is well known to produce relevant amounts of ^•^O in the gas and liquid phase [56, 57]. Without the O_2_ in the feed gas, one of the precursors of liquid ^•^NO would be missing, and in this condition no dissolved ^•^NO was detectable for both plasma jets by EPR.

In the presence of cysteine, no ^•^NO-adduct was detected. However, the absence of any ^•^NO-adduct signal does not necessarily mean that no ^•^NO is produced. Carboxy-PTIO reacts with ^•^NO to the imino nitroxide and ^•^NO_2_ (k_1_ = 0.5–16×10^4^ M^-1^ s^-1^) [58]. A faster reaction partner, *e*.*g*. low molecular weight thiols, may scavenge ^•^NO. The reaction rate of such a reaction was determined to be 1.5–3×10^5^ M^-1^ s^-1^, being slightly higher than the reaction of ^•^NO with Carboxy-PTIO and such result in its underestimation by EPR in the presence of cysteine [59].

**Fig 6:**
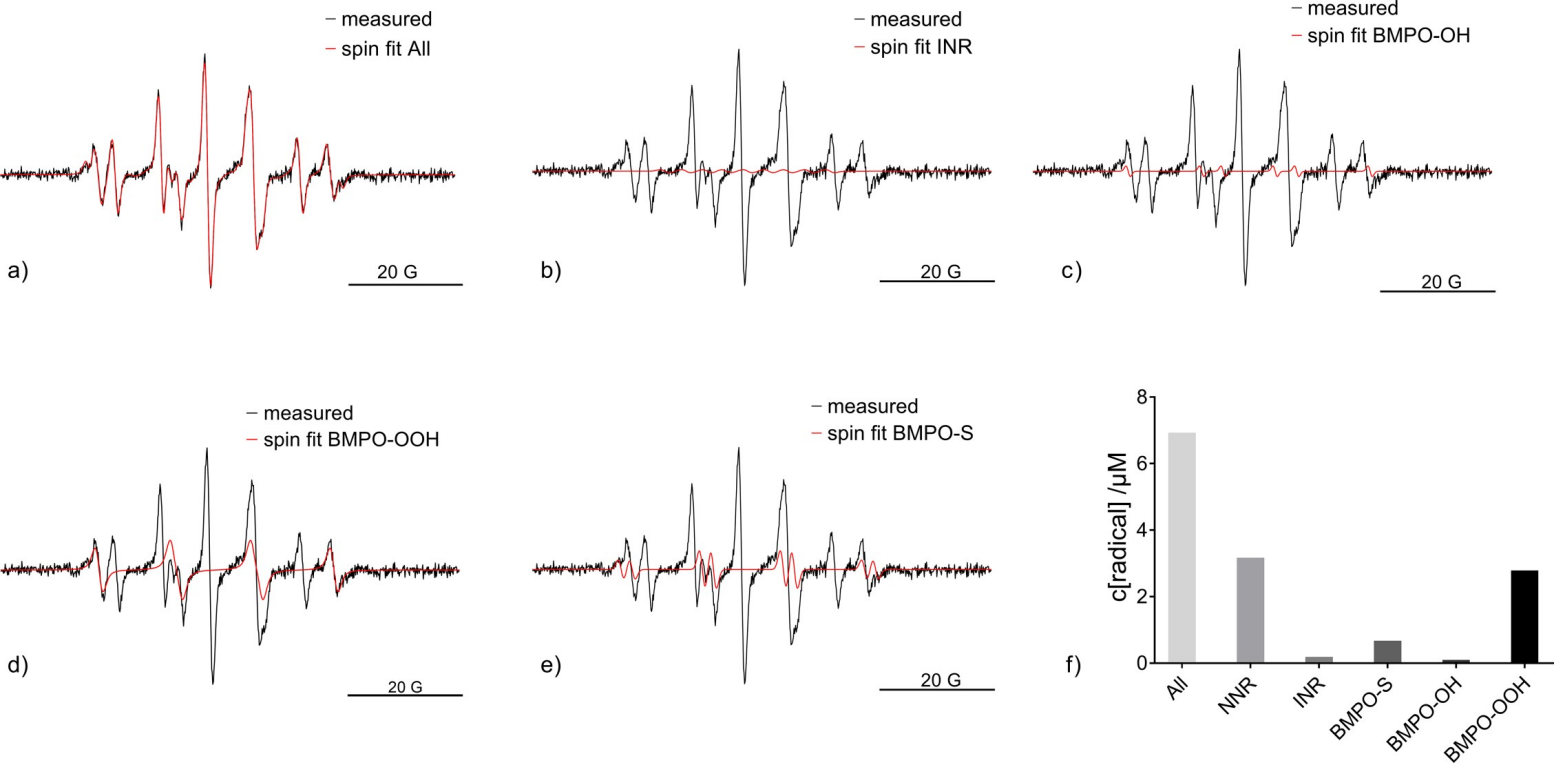
Measured and simulated EPR spectra after kINPen treatment. Measurements and fitted spectra are presented for (a) after treatment of ddH_2_O + cysteine with the kINPen with Ar + 0.7% O_2_ + 0.3% N_2_ and N_2_ curtain gas using both BMPO and Carboxy-PTIO as spin traps. In b), the measured spectra together with the ^•^NO-adduct (INR) are given. The measured spectra and simulated spectra of BMPO spin adducts of the hydroxyl (c), the superoxide anion (d), and the sulfur (e) radicals are shown (n = 1). In f), the resulting concentrations of the spin trap adducts after 30 seconds treatment of ddH_2_O+cysteine with the kINPen using N_2_ as curtain gas are given. The bars represent the overall radical concentration in the solution (all), NNR represents the concentration of the unreacted Carboxy-PTIO, INR of the ^•^NO–adduct, BMPO-S of the BMPO-trapped sulfur radicals, BMPO-OH of the BMPO-adduct of the hydroxyl radical, and BMPO-OOH of the BMPO-adducts of superoxide anion radicals.

The role of cysteine in altering NO detection via scavenging or other secondary effects was investigated by combining Carboxy-PTIO and BMPO to trap interfering radicals and measure these alongside ^•^NO. The measured EPR spectra together with the simulated sum spectra are shown in Fig 6A. Fig 6B shows the simulated spectra of the ^•^NO-adduct (INR), whereas Fig 6C–6E shows the adducts formed by BMPO: BMPO-OH (c), BMPO-OOH (d), BMPO-S (thiyl radical, e). The calculated concentrations of the spin adducts is given in Fig 6F. It needs to be kept in mind that all radicals have distinct reaction rates with the spin traps and different efficacies of being trapped.

Interestingly, by combination of the two spin traps, the ^•^NO-adduct became detectable again (Fig 6C). Moreover, also adducts of ^•^OH- and O_2_^•—^adducts of BMPO as well as a thiyl radical adduct of BMPO (BMPO-S) were detected. This demonstrates that the radicals interacting with cysteine hinder the detection of ^•^NO by Carboxy-PTIO. As soon as they were scavenged by BMPO, they were not able to interact with cysteine, and therefore, did not hinder the measurement any further. A potential underlying reaction pathway runs via hydroxyl radicals. Highly reactive ^•^OH is a likely candidate to attack the thiol group of cysteine [60], as the S-H bond energy is only 3.6 eV [61], yielding water and the detected thiyl radical. The latter can react with ^•^NO forming Cys-SNO, which is not EPR sensitive. Alternative reaction mechanism leading to S-H bond cleavage would have a similar effect [50]. Hence, it can be postulated that in the presence of thiyl radicals ^•^NO is scavenged to form Cys-SNO, evading detection by Carboxy-PTIO. For the COST-jet, the spin trap/cysteine mixture was performed as well. In contrast to the kINPen, this measurement only yielded the initial NNR signal and no BMPO-adducts or INR were detected after COST-jet treatment showing a complete absence of (detectable) ^•^NO (data not shown). This indicates that the ^•^NO-formation pathway is different for both plasma sources. Furthermore, the absence of ^•^OH and O_2_^•-^ indicates a different oxygen chemistry in the liquid as well. With that, the BMPO-S adduct was also missing as expected according to its formation by ^•^OH.

### Profile of the RNS reservoir (NO_2_^-^) and dump (NO_3_^-^)

To complement the analysis of short-lived species, the deposition of the reservoir RNS, nitrite (NO_2_^-^) and nitrate (NO_3_^-^) in presence or absence of cysteine was determined. Figs 8 (kINPen) and 7 (COST-jet) show the respective nitrite and nitrate profiles. For the kINPen, a large impact on deposition rate was found for the feed gas variation with the highest seen for O_2_ + N_2_ combination. The presence of cysteine modulated NO_2_^-^/NO_3_ deposition ratio, with higher amounts of NO_2_^-^ detected in its absence.

**Fig 7:**
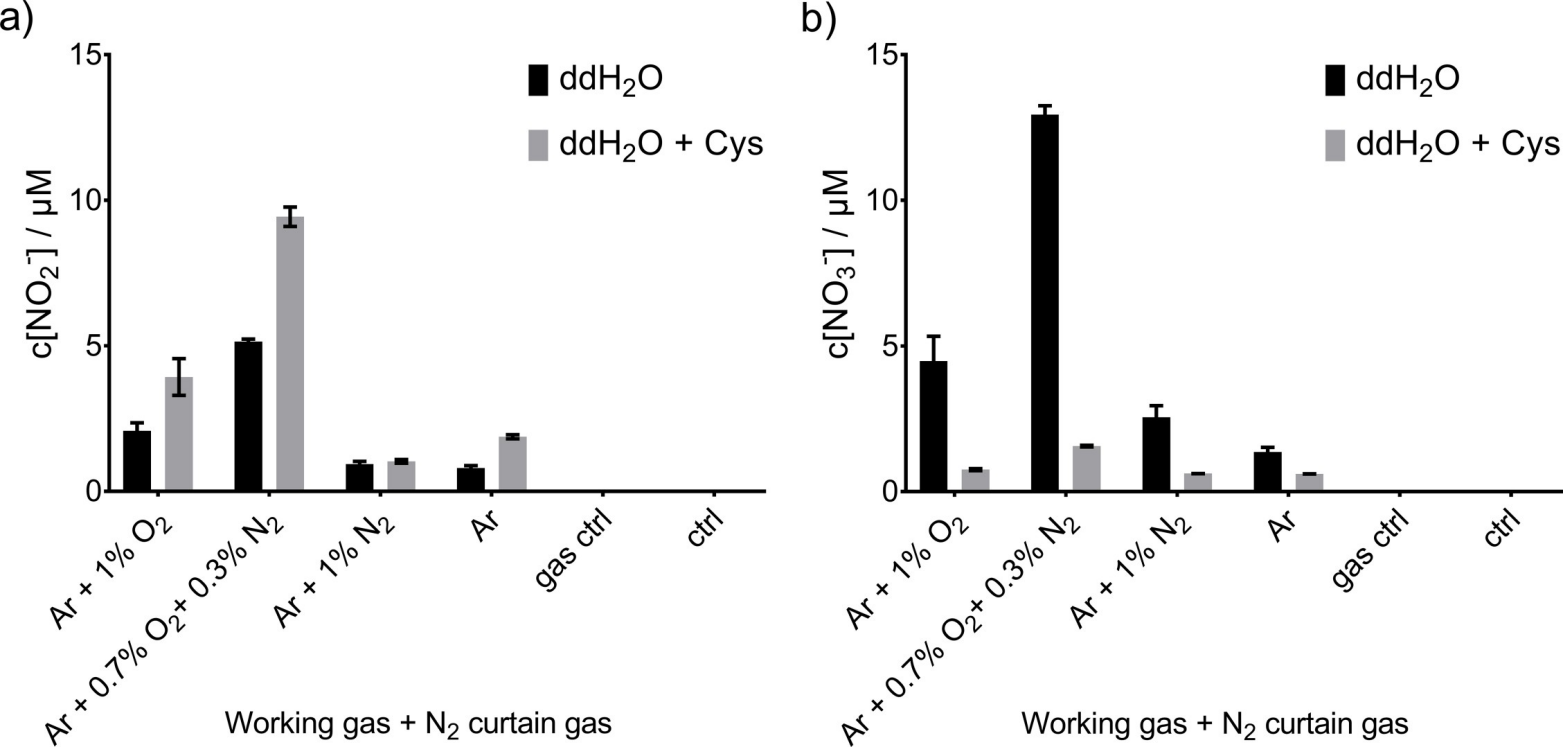
**NO**_**2**_^**-**^ **(a) and NO**_**3**_^**-**^ **(b) concentration after kINPen treatment**. kINPen was used with N_2_ curtain gas and with different feed gas compositions in a N_2_ environment provided by the curtain gas. Triplicates (n = 3) with standard deviation are shown.

**Fig 8:**
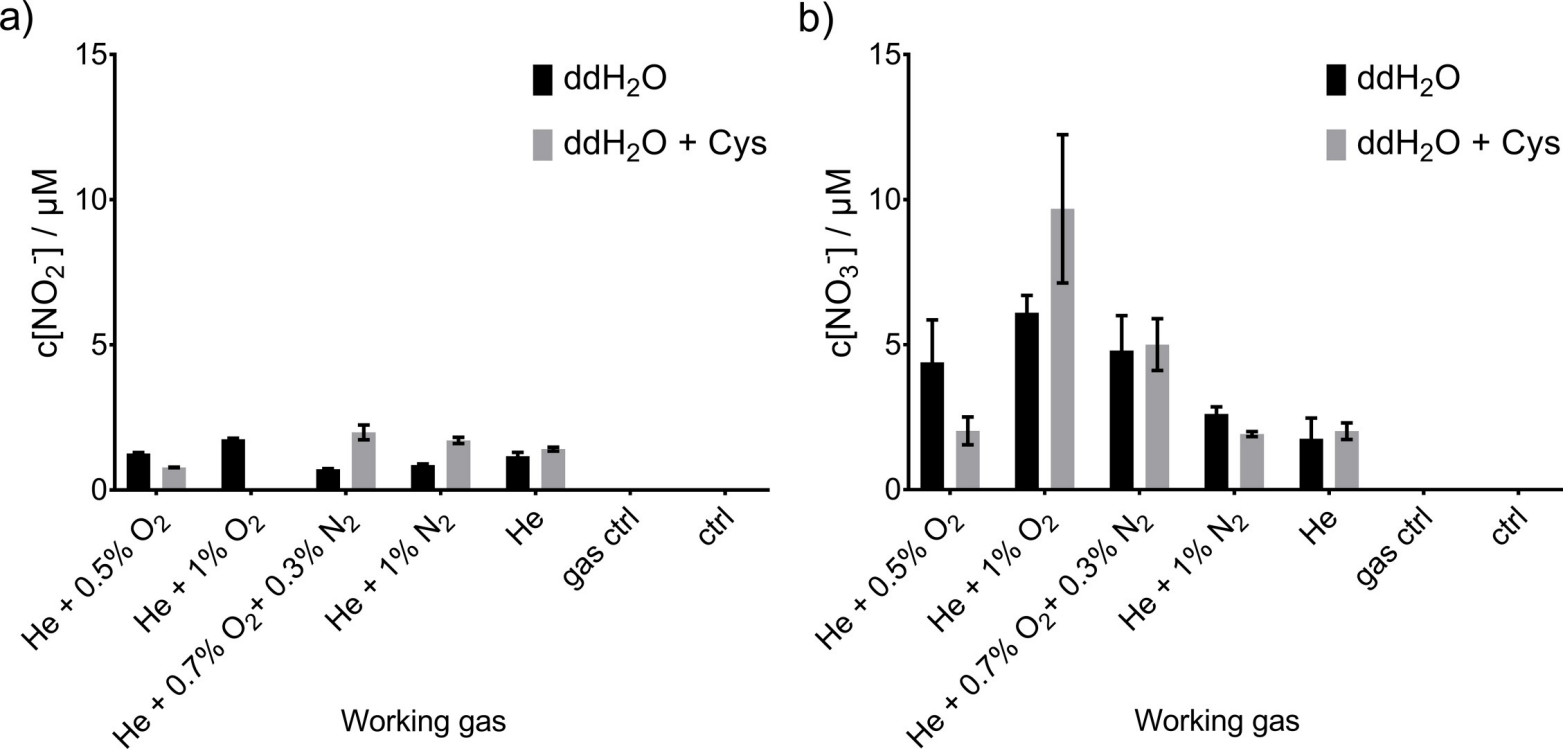
**Nitrite. NO**_**2**_^**-**^ **(a) and NO**_**3**_^**-**^ **(b) concentration after COST-jet treatment**. Treatment was performed in ambient air with the different feed gas compositions. Triplicates (n = 3) with standard deviations are shown.

In contrast, COST-jet treatment (Fig 7) resulted in lower and relatively equal levels of NO_2_^-^ with little impact of chosen feed gas composition. In the presence of cysteine, a slight trend following kINPen results could be observed. However, NO_3_^-^ production using the COST-jet was independent of cysteine presence in the treated solution. Most likely, the presence of ambient air around the effluent instead of nitrogen as for the kINPen, its different geometry and relative low gas flux contribute to the disparities. As already discussed above, the reaction pathways of RNS seem to be different from the kINPen. The stable production of low amounts of NO_2_^-^ and NO_3_^-^ under certain conditions (*e*.*g*. pure He or He + 1% N_2_) indicate that the observed species are not produced by secondary liquid reactions but predominantly by gas phase reactions. These species dissolve into the treated liquid and, given identical treatment times and gas flow pattern at the liquid surface, explain the rather invariable deposition. However, this notion also assumes a RNS gas phase chemistry that only slightly depends on feed gas composition and pointing towards the active He states.

After generation, further liquid chemistry affects (final) levels of NO_2_^-^ and NO_3_^-^. In ddH_2_O, deposited NO_2_^-^ can react further to ultimately yield NO_3_^-^ via two major pathways: either oxidation by H_2_O_2_ leading to the (transient) formation of peroxynitrite (ONOO^- -^) [62, 63] or disproportionation forming NO_2_ and ^•^NO [64], with subsequent disproportion of nitrogen dioxide and oxidation of ^•^NO by air or CAP derived oxygen species. A number of concurrent reactions allow a rapid rearrangement of the RNS in the liquid phase, with peroxynitrite as an important intermediate and hub and nitrate as the final, stable product [65]:

$$NO+O_{2}{}^{‑}\to ONOO^{‑},k=5\;x\;10^{9}M^{‑1}s^{‑1}$$ (1)

$$OH+NO_{2}+H_{2}O\to ONOO^{‑}+H_{3}O^{+}$$ (2)

$$2\;NO_{2}{}^{‑}+2\;H_{3}O^{+}\to NO_{2}+NO+3\;H_{2}O$$ (3)

$$2\;NO_{2}+3\;H_{2}O\to NO_{3}{}^{‑}+NO_{2}{}^{‑}+2\;H_{3}O^{+}$$ (4)

$$4\;NO+O_{2}+6\;H_{2}O\to 4\;NO_{2}{}^{‑}+4\;H_{3}O^{+}$$ (5)

$$4\;NO_{2}+O_{2}+6\;H_{2}O\to 4\;NO_{3}{}^{‑}+4\;H_{3}O^{+}$$ (6)

$$NO_{2}{}^{‑}+H_{2}O_{2}+H_{3}O^{+}\to NO_{3}{}^{‑}+H_{2}O+H_{3}O^{+}$$ (7)

$$NO_{2}{}^{‑}+H_{2}O_{2}+H_{3}O^{+}\to ONOOH+H_{2}O$$ (8)

$$ONOOH\to NO_{2}+OH$$ (9)

$$ONOOH+H_{2}O\to NO_{3}{}^{‑}+H_{3}O^{+}(80\%)$$ (10)

$$ONOOH+ONOO^{‑}+H_{2}O\to 2\;NO_{2}{}^{‑}+H_{3}O^{+}+O_{2}\left(20\%\right)$$ (11)

Both reaction pathways benefit from lower pH, and can be reduced via buffering at neutral to alkaline pH. Higher concentrations of NO_3_^-^ than NO_2_^-^ were expected in case of oxidizing conditions and cysteine, acting as a scavenger for oxidative species, subsequently increases the detected nitrite levels. Additionally, the NO_2_^-^ generated as a by-product by the direct reaction of the intermediates N_2_O_3_ or ONOO^-^ with cysteine to form S-nitrosocysteine may contribute to the observation [66].

### Deposition of hydrogen peroxide

**Fig 9:**
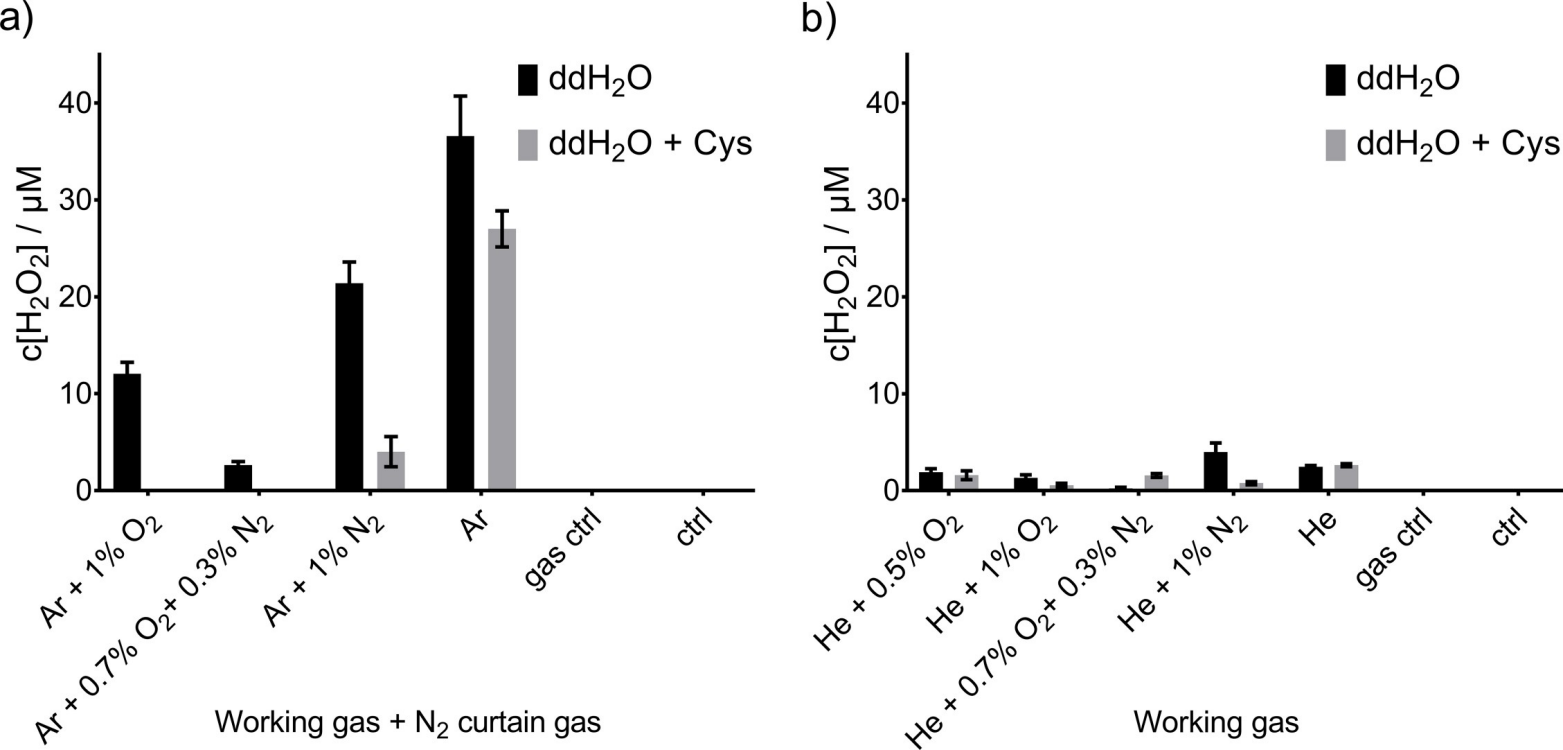
**Hydrogen peroxide concentration after plasma treatment using the kINPen (a) or COST-jet (b).** Treatment with the kINPen were performed in N_2_ environment due to curtain gas, COST-jet was operated in open air. Triplicates (n = 3) with standard deviation are shown.

Hydrogen peroxide (H_2_O_2_) is a long-lived reactive species known to affect various liquid chemistry reactions triggered by cold plasma treatment. The kINPen (Fig 9A) showed clear dependency of H_2_O_2_-production on the presence of cysteine and on the feed gas composition. The maximal H_2_O_2_ concentration was determined for pure argon feed gas (38 μM), with higher concentrations in ddH_2_O than for cysteine containing solutions. This was expected, as cysteine reacts with H_2_O_2_ forming predominantly cystine, with some other derivatives [50]. According to the observed product spectrum, it seems likely that H_2_O_2_ precursor compounds oxidize cysteine, which ultimately results in lower detectable H_2_O_2_ concentrations. Under dedicated conditions, one thiol moiety can react with up to five ^•^OH (one electron oxidants) to form a single cysteine sulfonic acid molecule [67]. Using the kINPen with oxygen admixture, no H_2_O_2_ was detectable in treated cysteine-containing solutions. Interestingly, these were the conditions where most ^•^NO was generated in the liquid and the highest production of Cys-SNO was observed. One reason could be that ^•^OH, a dominant precursor of H_2_O_2_, reacted directly with the cysteine, abstracting hydrogen and enabled ^•^NO and NO_2_^-^ to react directly with the cysteine and thereby being itself consumed.

For the COST-jet without cysteine present in the liquid, the highest concentration (5 μM) was reached for He + 1% N_2_, the other investigated conditions yielded similar concentration range with He + 1% O_2_ as lowest concentration reached (Fig 9B). Interestingly, the COST-jet seemed to be not so sensitively responding to molecular admixtures regarding the stable reactive oxygen and nitrogen species formation. A possible explanation might be the usage of the cold trap reducing the amount of water impurities, which most likely are the main source of H_2_O_2_, from the feed gas.

## Discussion

It is evident that the products derived from the plasma-assisted oxidation of cysteine mainly concern its thiol group, with the cysteine sulfonic acid and cysteine sulfinic acid as the main products even after short treatments. Cysteine sulfenic acid, which is a dominant precursor for downstream oxidation products, was not observed. According to the presence of cystine as a major product, it plays a role as intermediate. Assumingly its fast decay in the oxidative environment and further its instability during MS ionization contribute to this observation. These results are in good agreement with previous works conducted with a DBD, the COST-jet with other parameters, or the kINPen without curtain gas [26, 68]. Oxidation processes are the driving force under all tested conditions, suggesting that the plasma derived liquid chemistry in water is less variable than expected from gas phase measurements but seem to be strongly influenced by the chemical attributes of the presented target.

Besides the strongly oxidized products (sulfur oxidation state > + 2), cystine (oxidation state = −1) was highly abundant but further targeted by the plasma derived species with increasing treatment time to yield partially or fully oxidized disulfides (oxidation state = +1 and higher) [68]. As the targeted formation of disulfide bonds would allow the modulation of numerous cellular redox processes, shorter treatment times and/or less oxidizing conditions would allow control over the target instead of over-oxidation. However, experiments in more complex model systems as well as with other plasma sources are necessary to prove these points.

**Fig 10:**
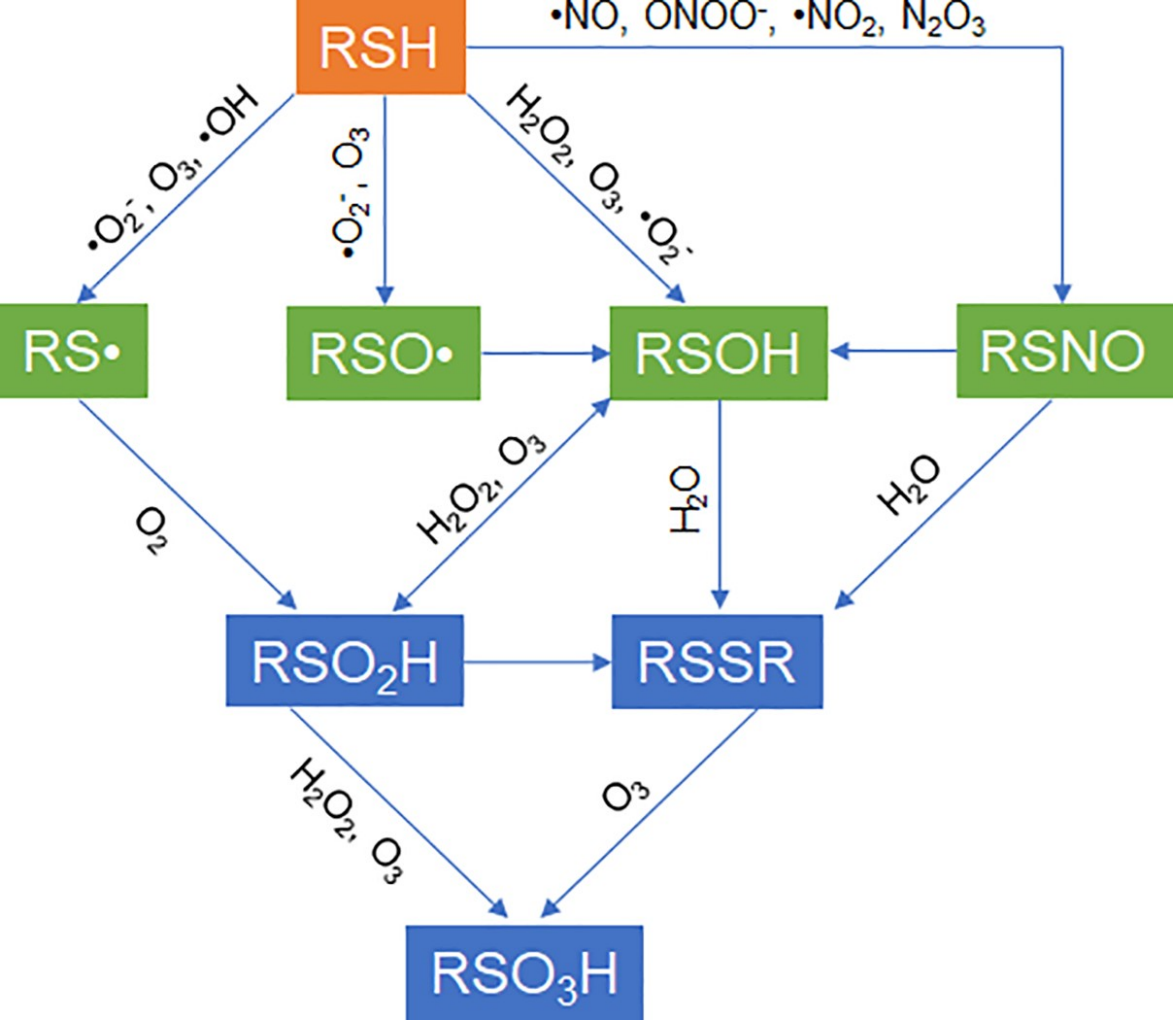
Possible pathway of formation of the main cysteine derivatives in oxidative cellular conditions. Cysteine (RSH, upper row) can react to the intermediary derivatives (middle row) cysteine sulfenic acid (RSOH), S-nitrosocysteine (RSNO), cysteinyl radicals (RS^•^) and cysteine sulfenyl radicals (RSO^•^). In turn, these react to the stable derivatives (lower two rows) cysteine sulfenic acid (RS_2_OH), cystine (RSSR), and cysteine sulfonic acid (RS_3_OH). Pathways taken from [80–83].

Besides a moderate target oxidation, an increased production of biologically active and clinically relevant S-nitrosocysteine directly at the location of interest and in combination with the other advantages of a plasma treatment would be highly beneficial in several pathologies including, *e*.*g*. chronic wounds [69–71]. Therefore, optimizing CAPs for the production of S-nitrosocysteine would open up additional medical treatment options as well as increase efficacies of current treatment regimes. However, with oxidative cysteine modifications making up the majority of products, formation of S-nitrosocysteine was observed to a limited extent only, even when discharge conditions were optimized for NO deposition (Fig 10). Compared to previous results [50], the usage of an N_2_ curtain combined with an admixture of 0.7% N_2_ + 0.3% O_2_ led to a threefold increase in Cys-SNO production compared to all other tested conditions. It seems likely that further tuning of the discharge parameters and ambient conditions could favor an increase in thiol nitrosylation. The low amounts detected may be attributed to a faster formation of the oxidative modifications and/or a fast decay of the nitrosylated thiols under the prevailing conditions. This is in line with formerly published data on nitrosothiol decay indicating an immediate decomposition in aqueous liquids, especially when the β-carbon atom carries an amino group yielding the corresponding disulfide (cystine) and nitric oxide [72]. Interestingly, the COST-jet was efficient at creating comparable amounts of Cys-SNO under all tested conditions, which is in good agreement with the observed working gas independent deposition of NO_2_^-^ and NO_3_^-^ and the likewise unaffected NO deposition as detected by EPR. It can be speculated that in the bulk liquid less oxidizing conditions than in kINPen prevail. Table 2 shows the qualitative performance of kINPen and COST jet sources as the working gas varies. Consequently, the production of reactive species (long and short-lived) and cysteine derivatives has been summarized here in order to compare the data and have an overall overview. In particular, it was possible to compare the two jets considering the fact that their qualitative performance fluctuates in the same qualitative production range.

**Table 2:** Qualitative overview of CAP performance using different working gases and for 30 seconds of treatment with kINPen (Ar-driven jet) and COST jet (He-driven jet).

	no treat.	Ar only	Ar/N_2_ 1%	Ar/N_2_/O_2_ 0.3/0.7%	Ar/O_2_ 1%	He only	He/N_2_ 1%	He/N_2_/O_2_ 0.3/0.7%	He/O_2_ 0.5%	He/O_2_ 1%
RSH^*^^a^	o^1^	− −	− −	− −	− − −	−	− −	− −	−	− − −
RSSR^*^^a^	o	+	+	+	++	o	o	+	+	o
**RSO**_**3**_H^*^^a^	o	+	++	++	+++	+	++	++	+	+++
**RSO**_**2**_H^*^^a^	o	++	++	++	++	++	++	++	++	+
RSNO^*^^a^	n.d.	+	+	+++	+	++	++	++	++	+
^**•**^NO^*^^d^	n.d.	n.d.	n.d.	n.d.	n.d.	n.d.	n.d.	n.d.	n.d.	n.d.
**NO**_**2**_^-^^*^^b^	o	+	o	+++	++	o	o	o	n.d.	o
**NO**_**3**_^-^^*^^b^	o	o	o	+	o	+	+	++	+	+++
**H**_**2**_**O**_2_^*^^c^	o	o	+	o	++	+	o	o	o	o
^**•**^**NO**°^**d**^	n.d.	n.d.	n.d.	++	+	n.d.	n.d.	++	+++	+++
**NO**_**2**_^**-**^°^**b**^	o	o	o	++	+	o	o	o	o	o
**NO**_**3**_^**-**^°^**b**^	o	+	+	+++	++	+	+	++	++	++
**H**_**2**_**O**_**2**_°^**c**^	o	++	++	+	+++	+	+	o	o	o

^a^ = MS quantification

^b^ = IC quantification

^c^ = FOX quantification

^d^ = EPR quantification

* = quantification with cysteine in solution

° = quantification without cysteine in solution

^1^ = relative abundance similar to control

n.d. = not determined

Several reaction pathways to yield nitrosylated thiols are described. In principle, a radical-driven formation is possible for direct plasma treatment. After an ^•^OH-induced H-abstraction at the thiol the resulting thiyl radical allows recombination with NO yielding the S-nitrosothiol. Although the formation of the thiyl radicals has been observed, this pathway may not contribute substantially as the number of species is low and the highly reactive OH radicals can attack all positions of the cysteine (radical). Further, at low pH (< 3) a direct addition of nitric oxide to the thiol can occur. However, this relatively inefficient reaction requires a 3^rd^ reaction partner to accept an excess electron [73]. In addition, while both COST-jet and kINPen are capable of pH reduction, the relatively large treated volume (750 μl) as well as the short treatment time (30 s) did not cause pH to drop to 3, thereby leaving direct Cys-SNO formation as a minor side reaction. Further pathways rely on nitric oxide oxidations products, such as ^•^NO_2_ and/or N_2_O_3_ [74]. At physiological pH, ^•^NO reacts with molecular oxygen forming principally ONOONO, a precursor of ^•^NO_2_ and/or N_2_O_3_, and the stable products NO_2_^-^, NO_3_^-^ [74]. It was shown that ^•^NO_2_ reacts with thiols to form the S-nitroso derivatives [74, 75]. A potentially major player of thiol nitrosylation is N_2_O_3_, which is formed by the following reaction [34]:

$$2\;{}^{•}NO+2\;NO_{2}^{-}+H^{+}\to 2\;N_{2}O_{3}$$ (12)

N_2_O_3_ in turn reacts with free thiol groups and can interact both with low molecular weight thiols, such as cysteine (2.6×10^−5^ M^-1^s^-1^) or glutathione (2.9×10^−5^ M^-1^s^-1^), and thiols in proteins (0.3×10^−5^ M^-1^s^-1^ for human serum albumin, all taken from [59]) by

$$RSH+N_{2}O_{3}\to RSNO+NO_{2}^{-}+H^{+}$$ (13)

The higher observed NO_2_^-^ concentrations in the presence of cysteine might partly be related to this reaction. Furthermore, future experiments will have to reveal if the chosen O_2_-N_2_ mixture in correlation with the N_2_ shielding might promote the direct formation of N_2_O_3_, thereby also circumventing the relatively slow formation of N_2_O_3_ in solution (~6.6×10^6^ M^-2^s^-1^, see [76]).

This would explain the higher amount of observed Cys-SNO after kINPen compared to COST-jet treatment, which seems to contradict EPR results showing larger amounts of the NO product INR after COST-jet exposure. However, it has to be kept in mind that the competing oxidative reactions, *e*.*g*. interaction with superoxide (around 10^2-3^ M^-1^s^-1^ as discussed in [77]) are significantly faster, thereby a total switch from oxidative to nitrosative conditions will not be possible. Under physiological conditions, nitrosoglutathione (GSNO) and S-nitrosothioredoxin levels regulate the formation of S-nitrosothiols, as they are responsible of the nitroso-group transfer, and indirectly, by the action of GSNO reductase [19]. This occurs as negative feedback for ^•^NO synthase and other downstream products of the pathway, *e*.*g*. the modified proteins [78, 79].

Cold physical plasma jets promise to be a valuable tool for optimizing the production and studying the action of bioactive compounds such as Cys-SNO. The obtained knowledge highlights a profound impact of cold plasma on protein thiol groups, with subsequent consequences in the cellular redox-signaling pathway including ^•^NO storage via nitrosylation. The question remaining is if optimized discharge conditions allow the production of sufficient amounts of Cys-SNO in complex environments to result in cellular responses, which have to be challenged in future work.

## Conclusions and outlook

The impact of an optimized treatment on the model target cysteine was compared to the COST reference jet using both standard conditions and adjusted molecular gas admixtures. Compared to a previous study using the kINPen without curtain gas, the usage of a curtain gas setup for the kINPen and modulation of the feed gas conditions increased the presence of nitrosative modifications on the thiol moiety of cysteine. While oxidative modifications still dominate the modification patterns, the first steps towards more nitrosative conditions are promising. Results are in good agreement with the observation of the ^•^NO-adduct, determined by EPR spectroscopy, and long-lived reactive species, namely H_2_O_2_, NO_2_^-^, and NO_3_^-^. It can be concluded that the general liquid chemistry induced by plasma treatment seems to be strongly influenced by the offered target even when changing atmospheric conditions to N_2_. Pending further investigations, it seems like the implementation of the curtain gas for certain clinical applications of CAPs might be a useful step to enhance the clinical potential of cold plasma treatment. Feed gas admixtures more readily modulated Cys-SNO production with active curtain gas compared to Cys-SNO production with comparable gas admixtures in ambient air.

Further studies will focus on using the COST-jet in a N_2_ atmosphere as well, as the ambient atmosphere seems to play a major role in RNS-driven liquid chemistry. Due to the much lower gas flux, a curtain gas system would cause much stronger distortion in case of the COST-jet, thereby the transfer of the whole setup into a controlled atmosphere seem more readily to facilitate. The impact can be expected to be less pronounced than with the kINPen as its discharge is not in direct contact with the ambient atmosphere In addition, usage of the COST-jet with its standard conditions allows a straightforward comparison to other sources using it as a reference to gain insight into similarities and differences between various plasma sources located in other labs. A critical step will be the implementation of stable isotope labels to elucidate the role of both gas and liquid phase chemistry. This would foster understanding if plasmas should be tuned specifically towards a desired gas chemistry or more towards initiating required liquid chemistry. Furthermore, translation of the results from the chemical model system into biological models is necessary to evaluate the predicted functional response.
